# Ultra-wideband polarization conversion metasurface and its application cases for antenna radiation enhancement and scattering suppression

**DOI:** 10.1038/s41598-017-16105-x

**Published:** 2017-11-23

**Authors:** Yuejun Zheng, Yulong Zhou, Jun Gao, Xiangyu Cao, Huanhuan Yang, Sijia Li, Liming Xu, Junxiang Lan, Liaori Jidi

**Affiliations:** 1 Information and Navigation College, Air Force Engineering University, Xi’an, 710077 China; 2 Science and Technology on Electronic Information Control Laboratory, Chendu, 610036 China

## Abstract

A double-layer complementary metasurface (MS) with ultra-wideband polarization conversion is presented. Then, we propose two application cases by applying the polarization conversion structures to aperture coupling patch antenna (ACPA). Due to the existence of air-filled gap of ACPA, air substrate and dielectric substrate are used to construct the double-layer MS. The polarization conversion bandwidth is broadened toward low-frequency range. Subsequently, two application cases of antenna are proposed and investigated. The simultaneous improvement of radiation and scattering performance of antenna is normally considered as a contradiction. Gratifyingly, the contradiction is addressed in these two application cases. According to different mechanism of scattering suppression (i.e., polarization conversion and phase cancellation), the polarization conversion structures are utilized to construct uniform and orthogonal arrangement configurations. And then, the configurations are integrated into ACPA and two different kinds of metasurface-based (MS-based) ACPA are formed. Radiation properties of the two MS-based ACPAs are improved by optimizing the uniform and orthogonal arrangement configurations. The measured results suggest that ultra-wideband polarization conversion properties of the MS are achieved and radiation enhancement and scattering suppression of the two MS-based ACPAs are obtained. These results demonstrate that we provide novel approach to design high-performance polarization conversion MS and MS-based devices.

## Introduction

Metasurfaces (MSs) are two-dimensional (2D) planar surfaces constructed by artificially periodic or quasi-periodic structures with sub-wavelength scales^1–6^, which overcome the physical limitations imposed by natural materials and provide exceptional capabilities for manipulating electromagnetic (EM) waves magnitudes^7,8^, phases^9–11^, polarizations^12,13^, propagation directions^14–16^ and shapes^17,18^. Recently, polarization conversion was one of the most interesting fields of MSs. There are two major research interests on the polarization conversion MSs. One is to realize almost arbitrary polarizations from one specific state to another. Linear-to-linear^19,20^, linear-to-circular^21,22^ and circular-to-circular^23^ polarization converters were presented, which provided flexible control of polarizations. The second research interest is to broaden polarization conversion bandwidth. Structure optimization^24^, topology optimization^25^ and multiple layers cascading^26^ were demonstrated to be effective bandwidth broadening methods. However, the present bandwidth broadening methods were mostly toward high-frequency range. The ultra-wideband polarization conversion MS, which covers low-frequency band, is rarely reported.

Because of the excellent characteristics of polarization conversion MSs, a series of exotic applications were carried out^27–34^, especially in antenna field^35–41^. Two transmitted polarization conversion MSs were employed to patch antenna and horn antenna, respectively^35^. The linearly polarized radiant waves were converted to circularly polarized waves. Except for converting the antenna polarizations, the polarization conversion structures were used to suppress antenna scattering. These polarization conversion structures were used to form orthogonal arrangement configurations and applied to patch antenna and slot array antenna directly^39,40^. Radar cross-section (RCS) of the antennas was reduced based on phase cancellation principle. In the above designs, radiation properties of the antennas were preserved. Best operation bandwidth of the MS-based antenna was 8.0%. Applying the polarization conversion structures to a wideband antenna and improve the radiation and scattering performance simultaneously is rarely presented in existing literatures.

In this paper, we present a double-layer complementary MS with ultra-wideband polarization conversion bandwidth. Then, we propose two application cases by applying polarization conversion structures to aperture coupling patch antenna (ACPA). Due to the existence of air-filled gap of ACPA, air substrate and dielectric substrate are used to form double-layer MS. The complementary anisotropic patterns are printed on both sides of the dielectric substrate. The polarization conversion bandwidth is broadened toward low-frequency range for the introduction of air substrate. Thus, ultra-wideband polarization conversion bandwidth is achieved. Subsequently, according to different mechanism of scattering suppression, the polarization conversion structures are used to construct uniform and orthogonal arrangement configurations. Referring to previous loading methods^38–42^, the configurations could be readily integrated into ACPA. The loaded configurations are optimized to obtain better impedance matching and radiation performance. The two MS-based ACPAs possess radiation enhancement and scattering suppression simultaneously. The measured results demonstrate the validity of our design methods.

## Results

### Design and analysis of polarization conversion metasurface

**Figure 1:**
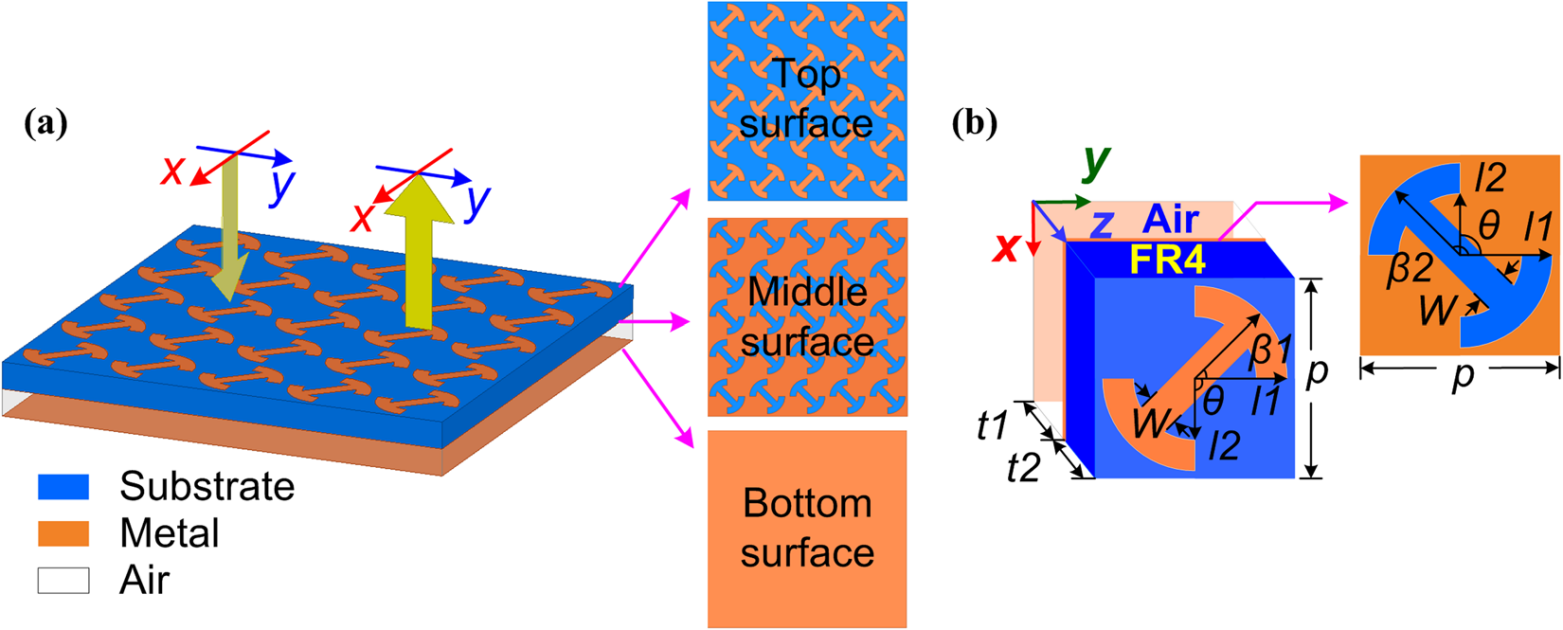
Schematic illustration of (**a**) proposed polarization conversion MS under the illumination of linearly polarized waves and (**b**) 3D schematic of a unit cell. Polytef dielectric substrate and air substrate are utilized to form this double-layer structure. The relative permittivity of polytef dielectric substrate is 2.65 and dielectric loss is 0.002. The design patterns are printed on both sides of dielectric substrate. The detailed dimensions of unit cell are *p* = 10.0 mm, *l1* = 4.4 mm, *l2* = 2.8 mm, *w* = 1.6 mm, *t1* = 3.0 mm, *t2* = 3.0 mm, *θ* = 90°, *β1* = 45°/135°, *β2* = 135°/45°.

Fig. 1(a) presents the schematic illustration of proposed double-layer polarization conversion MS illuminated by *x*-polarized EM waves. The MS converts polarization state to its orthogonal counterpart in an ultra-wide frequency range. Fig. 1(b) gives the 3D schematic structure of a unit cell. Metallic ground plane on the bottom surface is installed to guarantee absolute reflection. Air substrate and dielectric substrate are used to form double-layer structure. The dielectric substrate is on the above. The thicknesses of them are *t1* and *t2*, respectively. Polytef slab (FR4) is adopted as dielectric substrate. The relative permittivity is 2.65 and dielectric loss is 0.002. Modified I-shaped patch and its orthogonal complementary slot are printed on the top and bottom surfaces of dielectric substrate, respectively. All parameters of the unit structures are optimized to obtain desired characteristics.

**Figure 2:**
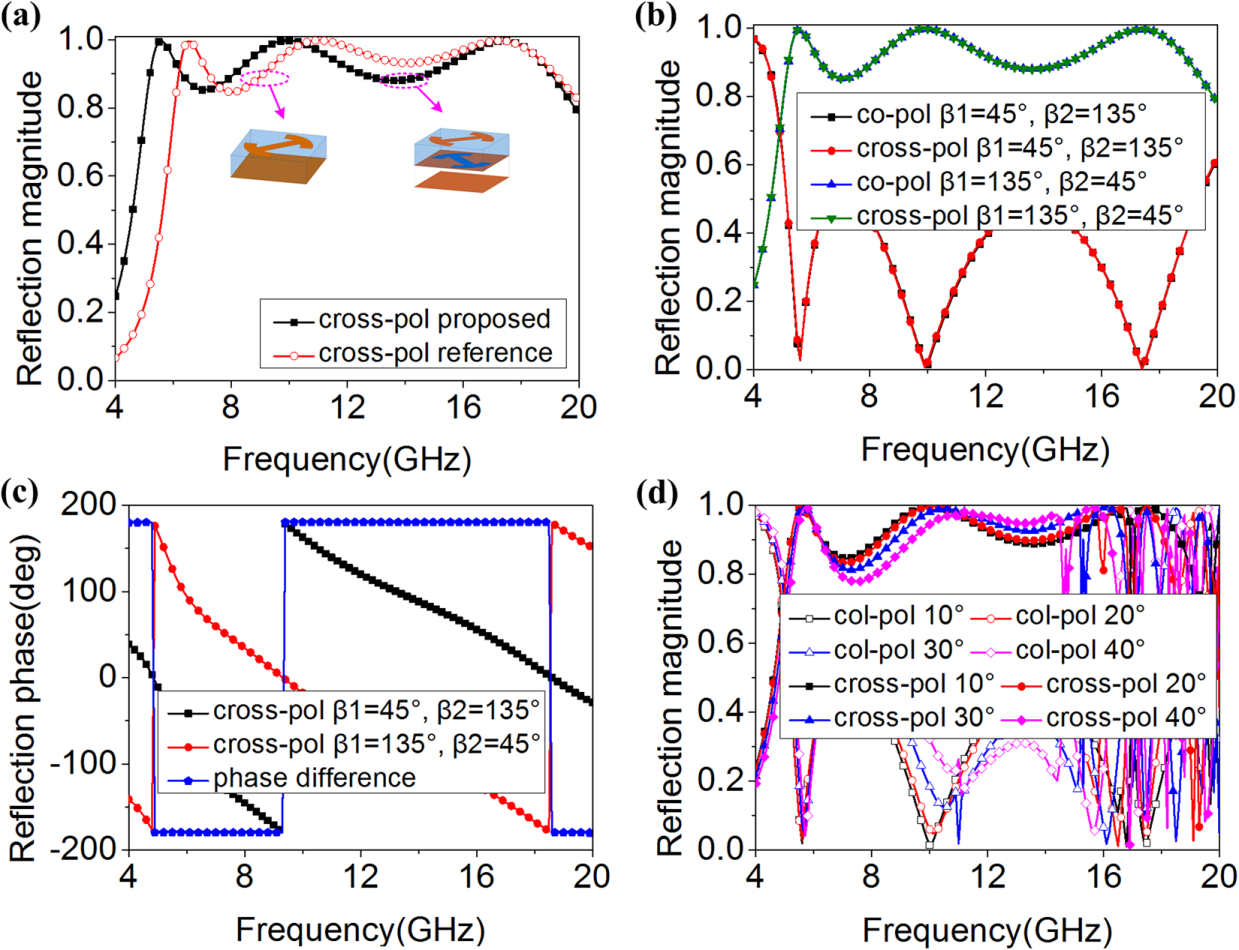
(**a**) Comparison of reflection magnitudes of single-layer reference unit and double-layer proposed unit. (**b,c**) Reflection magnitudes and phases of proposed units for different *β1* and *β2* under normal *x*-polarized incident waves. (**d**) Reflection magnitudes of proposed units for different oblique incident angles.

To investigate reflection properties of the proposed unit, full-wave numerical analysis was accomplished in commercial software HFSS with master-slave periodic boundaries in *x* and *y* directions and floquet ports in *z* direction. As Fig. 2(a) shows, a single layer unit structure with modified I-shaped patch is taken as a reference (i.e., as a comparison). Polarization conversion bandwidth of proposed unit is broadened toward low-frequency range. Moreover, the reflection magnitudes are kept well although the bandwidth is broadened. Most incident waves are reflected to its orthogonal counterparts. Taking a further step, the reflection magnitudes and phases of proposed unit and its mirror one (i.e., the proposed unit rotates 90°) are investigated in detail for normal incidence. The reflection magnitudes of the mirror unit coincide well with the proposed one, presented in Fig. 2(b). The curve of cross-polarization shows three peak values at 5.6, 9.9 and 17.4 GHz, where the incident waves are effectively converted to the cross-polarized one. Moreover, the values of reflection magnitudes are more than 0.85 from 5.1 to 19.5 GHz. As Fig. 2(c) shows, reflection phase difference is equal to 180° between the proposed unit and its mirror one, which satisfies the condition of phase cancellation principle. For oblique incidence, the reflection magnitudes of the proposed unit are shown in Fig. 2(d). With incident angles increase to 40°, the polarization convention properties are almost maintained from 4.0 to 15.0 GHz.

**Figure 3:**
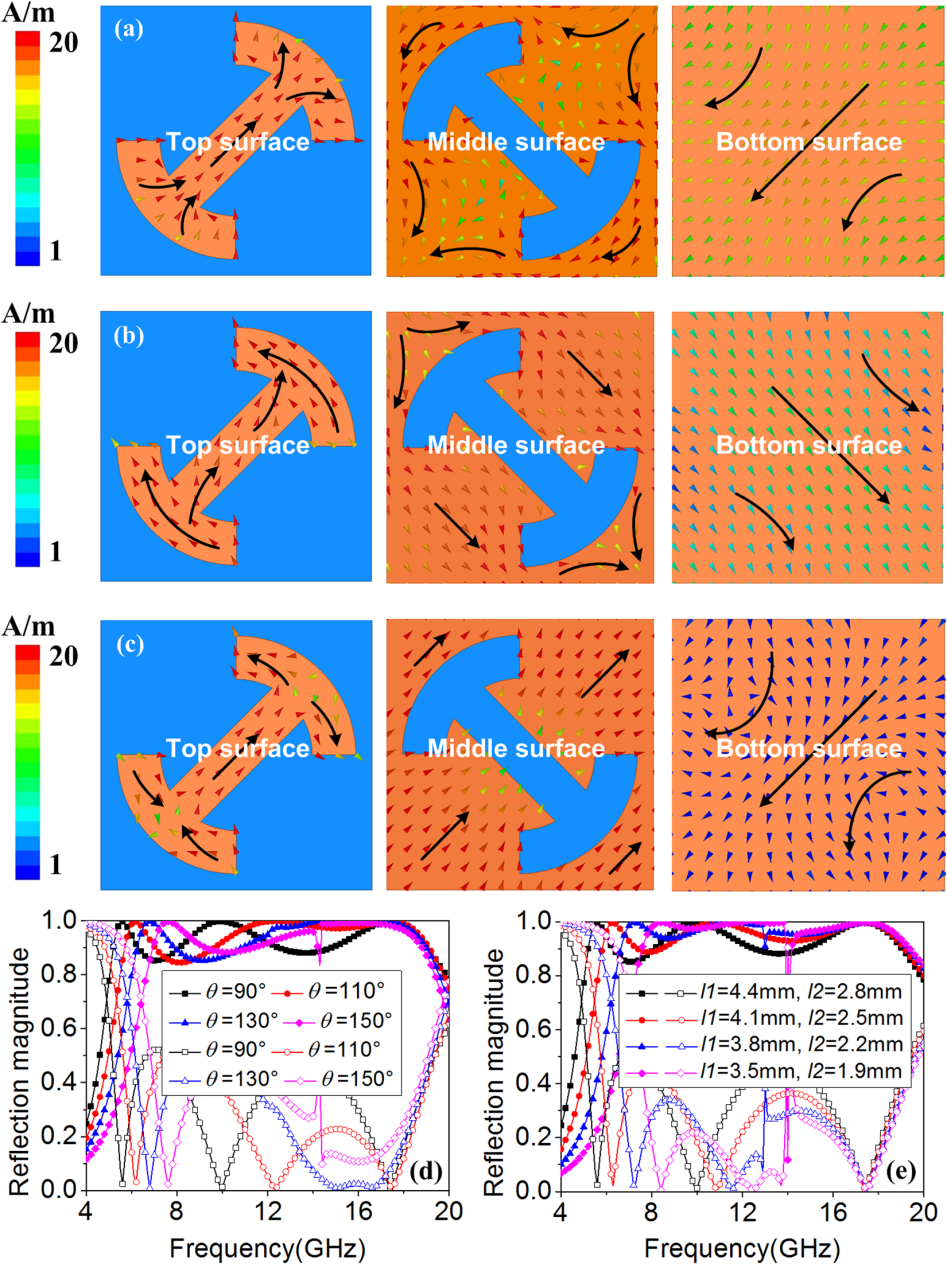
Current distributions of proposed units at (**a**) 5.6 GHz, (**b**) 9.9 GHz and **(c)** 17.4 GHz. Reflection magnitudes for different (**d**) *θ* and (**e**) *l1* and *l2* under normal *x*-polarized incident waves.

To further reveal operation mechanism of proposed unit, current distributions of proposed unit at 5.6, 9.9 and 17.4 GHz (peak reflection magnitude frequency) were investigated. Intense induced current is observed on the top and middle surfaces at three frequencies, presented in Fig. 3. As Fig. 3(a) shows, the current directions on the top surface are completely different from those on the middle surface. Strong interaction is produced between the top and middle surfaces. However, the different current directions are mainly observed on the “−” of modified I-shaped patch, presented in Fig. 3(b). Strong interaction is produced between the “−” of modified I-shaped patch and middle surface. Furthermore, as Fig. 3(c) plots, the different current directions are mainly observed the “−” of I-shaped patch itself. Strong interaction is produced between the “−” of I-shaped patch itself. It can be concluded that the interaction between the top and middle surfaces becomes weak with the increase of frequency. The similar phenomena are observed on the metallic ground plane. Although the current directions are different between top and bottom surfaces at 5.6, 9.9 and 17.4 GHz, the strong interaction is mainly produced at 5.6 GHz. Thus, polarization conversion bandwidth is broadened toward low-frequency range.

To verify the above analysis, parameters investigations were conducted. As Fig. 3(d) shows, with the increase of *θ* (the length of “−” of modified I-shaped patch is decreased), the peak values of cross-polarization at 5.6 GHz and 9.9 GHz shift toward high-frequency range, whereas the peak value at 17.4 GHz shift slightly toward low-frequency range. Moreover, the peak values of cross-polarization at 9.9 GHz and 17.4 GHz are overlapped when *θ* equal to 150°. That is to say, the peak value at 17.4 GHz disappears. Taking a further step, the reflection magnitudes for different *l1* and *l2* are shown in Fig. 3(e). With the decrease of *l1* and *l2*, the peak values of cross-polarization at 5.6 GHz and 9.9 GHz shift toward high-frequency range while the peak value at 17.4 GHz is almost maintained. That is to say, the peak values at 5.6 GHz and 9.9 GHz are influenced when the “−” and “|” of modified I-shaped patch are decreased. Because the peak values of cross-polarization at 17.4 GHz is caused by I-shaped patch itself, the interaction between the two “−” structures of modified I-shaped patch becomes strong when the length of two “|” structures are decreased. Thus, the peak value at 17.4 GHz is almost maintained. From above parameters investigations, the current distributions analysis of proposed structures is reasonable.

### Application cases of metasurface-based aperture coupling patch antenna

Although more and more novel characteristics of MS were reported, such as polarization conversion, the application of MS is still a challenge by using these novel properties. With the development of stealth technology, high-performance EM devices are urgently required. Thus, in this article, we implement an academic study and propose two application cases of MS-based antenna by using the designed MS.

**Figure 4:**
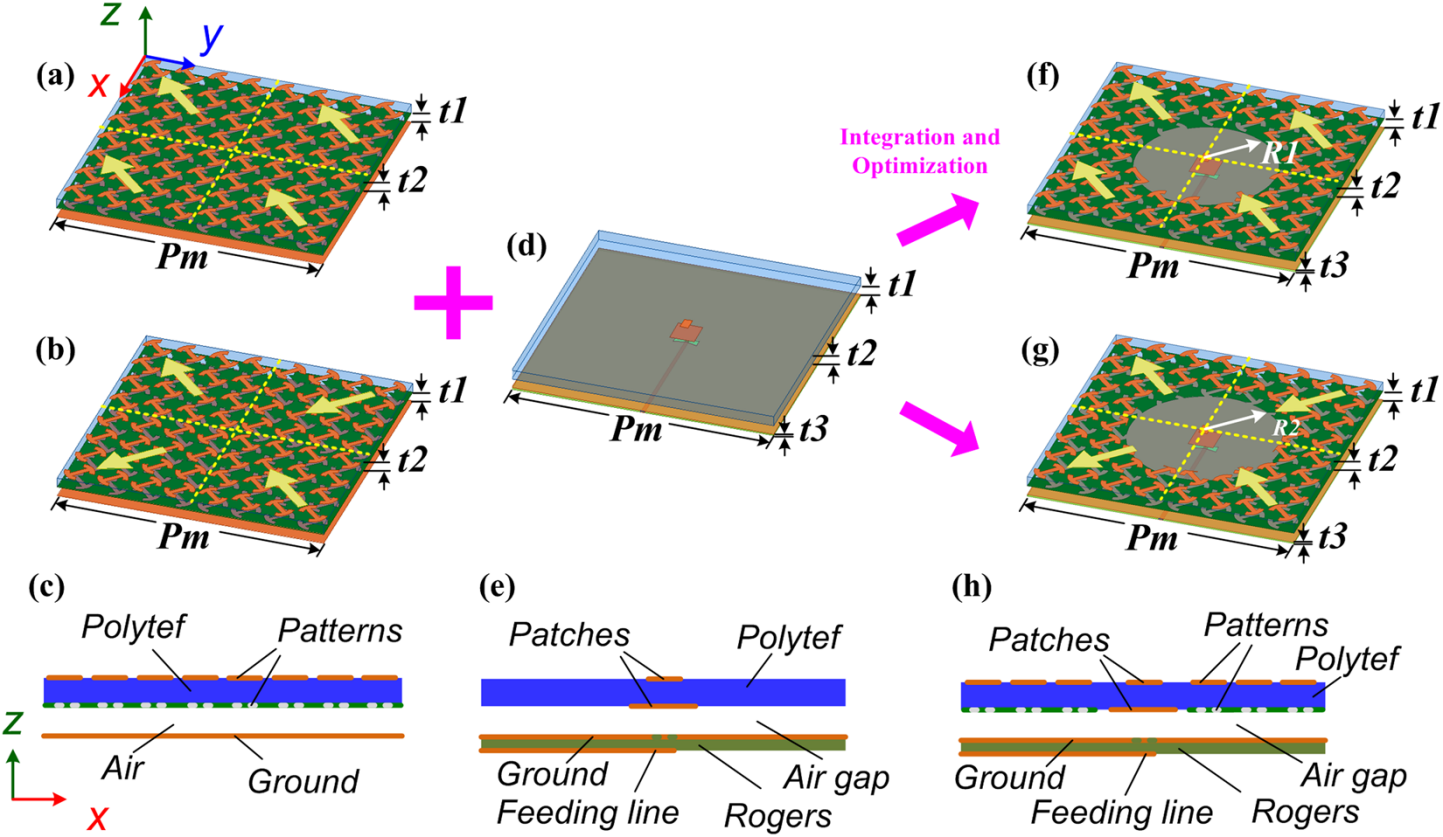
Schematic view of two different application cases. 3D view of (**a**) polarization conversion MS with uniform arrangement (denoted as PCS#1) and (**b**) polarization conversion MS with orthogonal arrangement (denoted as PCS#2). (**c**) Side view of those kinds of MSs. (**d**) 3D view of primary ACPA. (**e**) Side view of ACPA. 3D view of (**f**) proposed PCS#1 antenna and (**g**) proposed PCS#2 antenna. (**h**) Side view of those kinds of antennas. The length *Pm* = 80.0 mm. The thickness *t1* = 3.0 mm, *t2* = 3.0 mm, and *t3* = 0.508 mm.The radii of circular-shaped areas of proposed PCS#1 and PCS#2 antennas are *R1* = 20.7 mm, *R2* = 22.5 mm, respectively.

**Figure 5:**
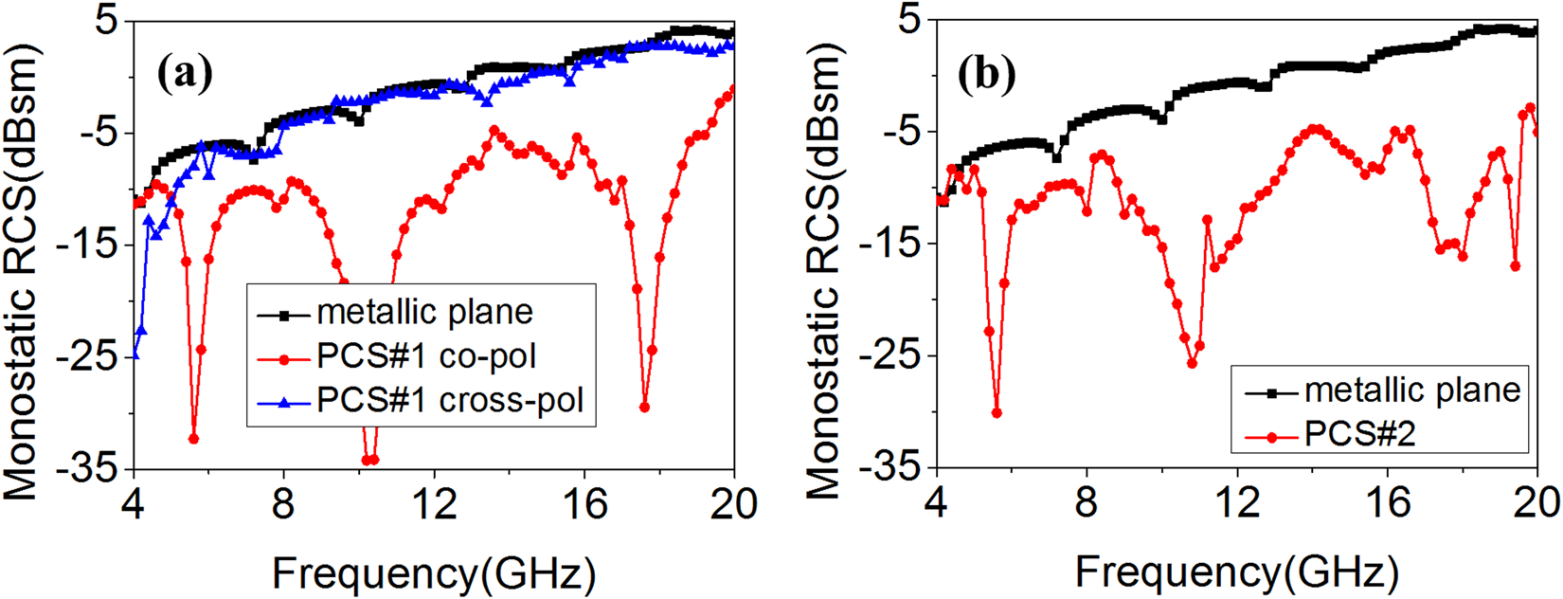
Scattering suppression of the MS. (**a**) PCS#1 and (**b**) PCS#2 for normal *x*-polarized incident waves.

Firstly, two different kinds of MSs were constructed by using the designed polarization conversion structures. Top and side views of the MSs are shown in Fig. 4(a–c). To maintain the characteristics of the structures, each MS consists of 2 × 2 tiles, and each tile is comprised of 4 × 4 polarization conversion units. The difference is the arrangement of these tiles. As Fig. 4(a) presents, the polarization conversion tiles are arranged in uniform configuration and the scattering suppression could be produced based on polarization conversion principle. This kind of MS is denoted as PCS#1. Meanwhile, the polarization conversion tiles are arranged in orthogonal configuration, depicted in Fig. 4(b), and the scattering suppression could be produced based on phase cancellation principle. This kind of MS is denoted as PCS#2. To verify the scattering performance of PCS#1 and PCS#2, full-wave numerical analysis was carried out in Ansoft HFSS. As Fig. 5 shows, for normal incidence, both PCS#1 and PCS#2 possess scattering suppression almost from 4.0 to 20.0 GHz.

As promising application of MS, aperture coupling patch antenna (ACPA) was selected as primary antenna for its low-profile, easy-feed and broad operation band, shown in Fig. 4(d,e). Feed line and ground plane are printed on the top and bottom surfaces of Rogers RT/duroid 4350 substrate (ε = 3.48, tanδ = 0.003) with a thickness of *t3*. Rectangular parasitic patches are etched on the both sides of Polytef slab (*ε* = 2.65, tan*δ* = 0.002). The thickness of this substrate is *t2*. The parasitic patches are coupled with the feed line through a papilionaceous slot in the ground plane, and it is spaced from the ground plane by an air-gap. The thickness of the air-gap structure (i.e., air substrate) is *t1*. Same Polytef substrate, air substrate and ground plane of the MSs and ACPA are observed. Thus, the designed MSs could be readily integrated into ACPA and MS-based antennas are constructed, shown in Fig. 4(f–h). The antenna shown in Fig. 4(f) is denoted as proposed PCS#1 antenna and the other one shown in Fig. 4(g) is denoted as proposed PCS#2 antenna. The polarization conversion structures located at the central area are removed to place radiation parasitic patches. To preserve impedance matching and obtain enhanced radiation performance, a circular-shaped area of polarization conversion structures is removed. The radius of the removed area of each MS-based antenna is optimized, respectively. The selection criteria of optimum radii should properly consider the following four factors: 1) the impedance matching and operation band of antenna should be maintained; 2) the gain of antenna could be enhanced in the whole operation band as far as possible, then the gain should be as large as possible; 3) To avoid making much impact on scattering performance of PCS#1 or PCS#2, the radii of circular-shaped areas should be selected as small as possible; 4) chipped structures should not be observed when the circular-shaped areas are removed, it may cause much fabrication tolerance. The MS-based antennas possess two different types because they are integrated with different MSs. As a comparison, the reference single-layer MSs are also applied to ACPA. The radii of circular-shaped areas of the reference MS-based antennas are equal to those of proposed MS-based antennas. Different form the proposed MS-based antennas, the bottom surface of Polytef substrate of reference MS-based antennas are metallic plane. The reference antennas are denoted as reference PCS#1 antenna and reference PCS#2 antenna, respectively.

**Figure 6:**
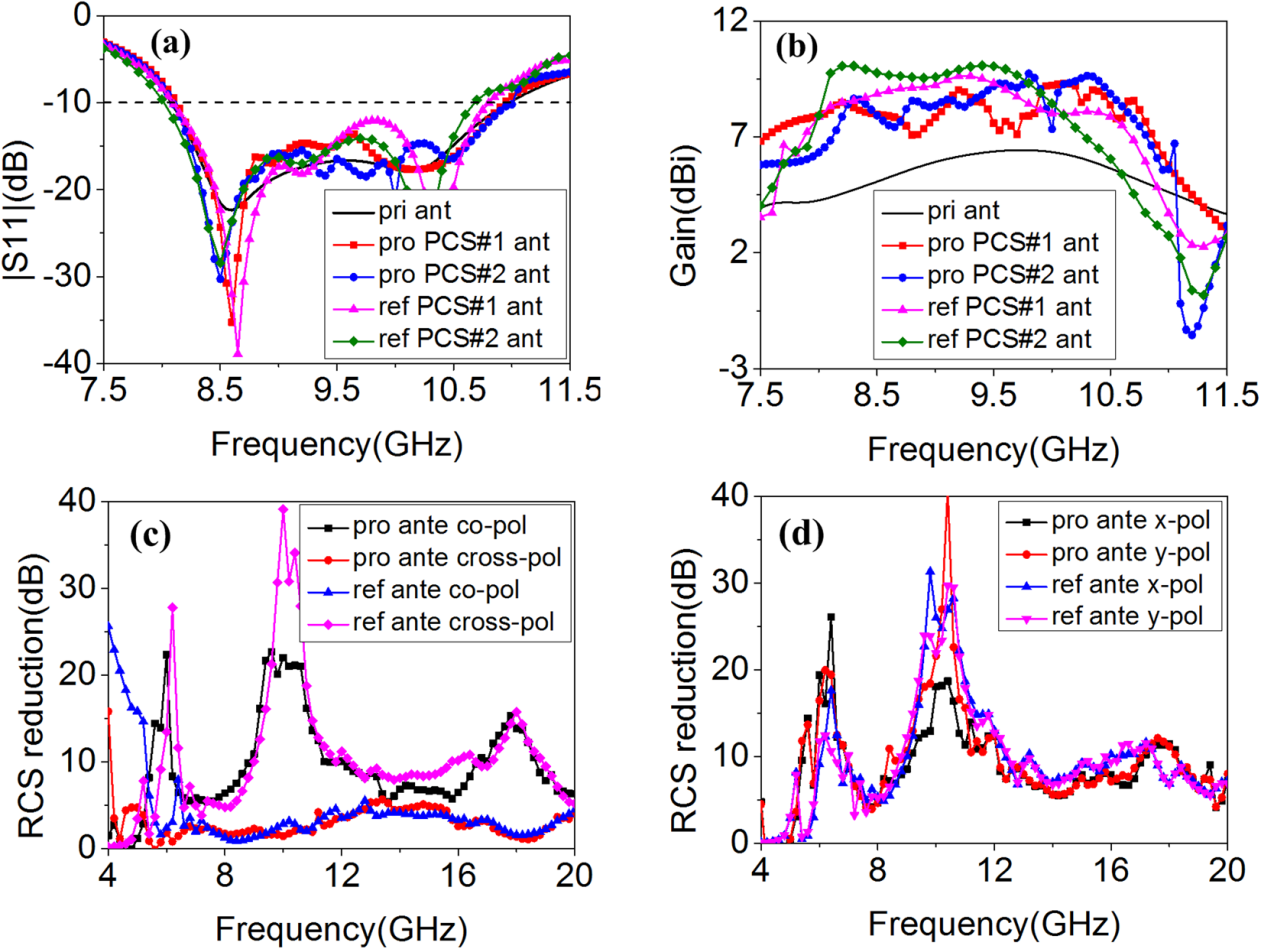
Radiation and scattering properties comparison of single-layer reference antennas and double-layer proposed antennas. (**a**) S_11_, (**b**) Boresight gain, (**c**) RCS reduction of reference and proposed PCS#1 antenna for normal *x*-polarized incident waves. (**d**) RCS reduction of reference and proposed PCS#2 antenna under normal *x*- and *y*-polarized incidence.

To demonstrate the validity of our designs, radiation and scattering properties of the antennas were investigated. The S_11_ of the antennas are presented in Fig. 6(a). Impedance matching and operation bandwidth of proposed MS-based antennas (8.0~11.0 GHz) are maintained well. The operation band of reference MS-based antennas is slightly narrowed. The boresight gain of those antennas is plotted in Fig. 6(b). Remarkable improvements are observed in a wide frequency range which covers its whole operation band. The gain enhancement bandwidth of proposed MS-based antennas is broader than that of reference MS-based antennas. Because of the different mechanism of scattering suppression, scattering performance of the four MS-based antennas was investigated, respectively. As Fig. 6(c) shows, when *x*-polarized waves are normally illuminated on the proposed and reference MS-based PCS#1 antennas, the polarization of substantial components of the scattered field are orthogonal to those of the incident waves. Thus, co-polarized scattering is suppressed remarkably based on polarization conversion characteristics. Three peak values of scattering suppression are observed. The frequencies of peak scattering suppression almost coincide with those of best polarization conversion. Compared with proposed or reference PCS#1 antenna, the polarization conversion tiles on first and third quadrant of proposed or reference PCS#2 antenna are rotated 90°. As discussed previously, the reflection magnitudes of cross-polarized waves are not influenced while the reflection phases are changed. Figure 6(d) presents monostatic RCS reduction for normal *x*- and *y*-polarized incident waves. The scattering is suppressed for arbitrary polarizations based on phase cancellation principle. Meanwhile, as Fig. 6(c,d) show, scattering suppression bandwidth of proposed MS-based antennas is broadened toward low-frequency range compared with that of reference MS-based antennas. From above results, proposed MS-based antennas possess better radiation and scattering performance.

**Figure 7:**
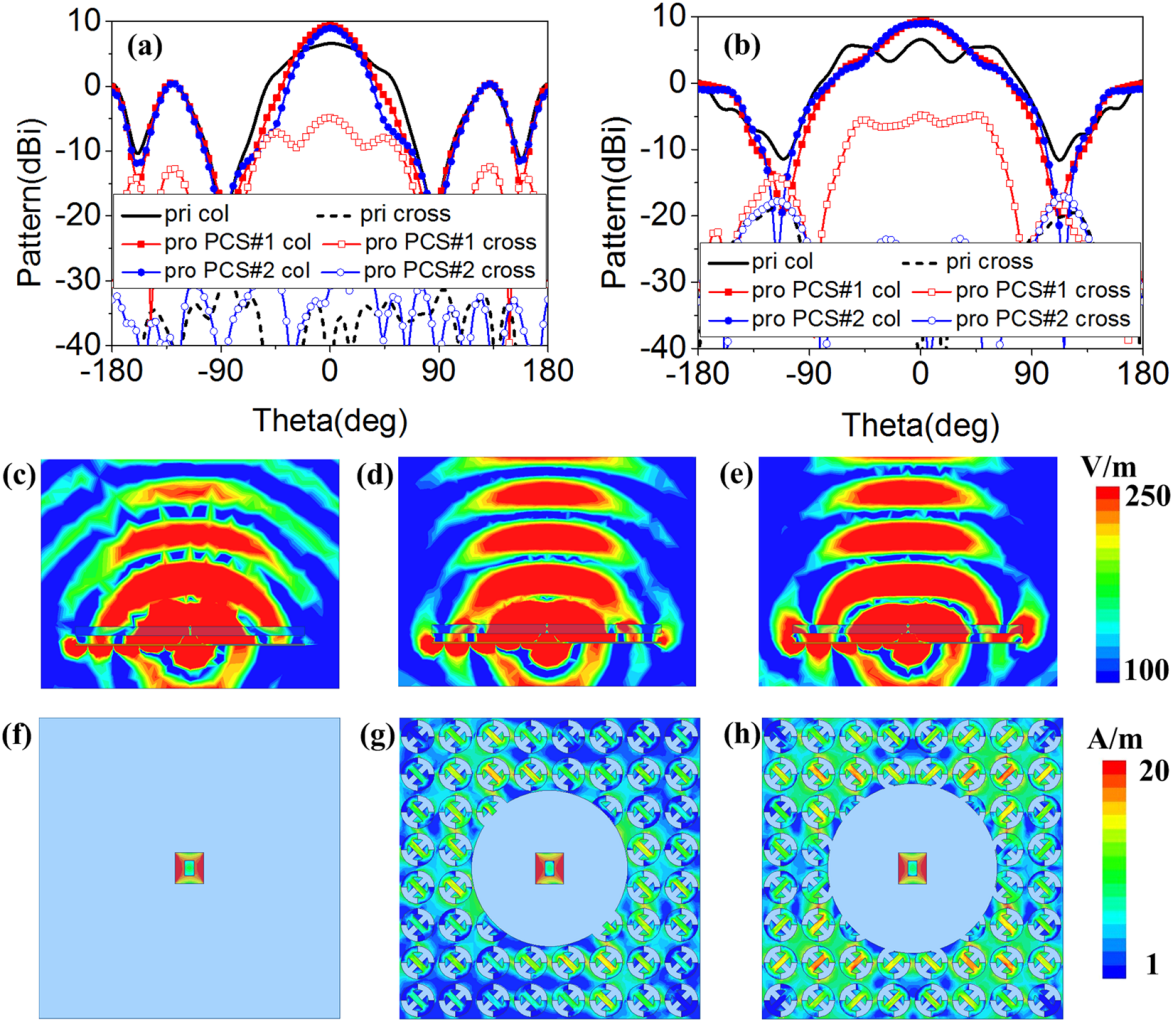
(**a**) x*oz* and (**b**) *yoz* plane radiation patterns at 10.1 GHz. Electric fields of (**c**) primary antenna, (**d**) proposed PCS#1 antenna and (**e**) proposed PCS#2 antenna at 10.1 GHz. Current distributions of (**f**) primary antenna, (**g**) proposed PCS#1 antenna and (**h**) proposed PCS#2 antenna at 10.1 GHz.

To further investigate the radiation properties of proposed MS-based antennas, the radiation patterns comparison of primary and proposed antennas at 10.1 GHz (proposed PCS#1 and PCS#2 antennas possess the same gain) are depicted in Fig. 7(a,b). Similar radiation patterns of proposed PCS#1 and PCS#2 antennas are observed. The main beams of *xoz* and *yoz* plane are narrowed. Therefore, the boresight gain is enhanced. In addition, the values of cross-polarization of proposed PCS#1 and PCS#2 antennas are less than –15 dB and –27 dB, respectively. It can be concluded that the radiation properties of proposed antennas are improved. To reveal the reason of radiation enhancement, electric fields and current distributions of primary and proposed antennas are investigated. As Fig. 7(c–e) shows, similar electric fields of proposed antennas are observed which is stronger than those of primary antenna. Moreover, the spherical waves of primary antenna are transformed to approximated plane waves of proposed antennas. The radiation waves are focused. Taking a further step, the current distributions are shown in Fig. 7(f–h). Intense current is induced on polarization conversion structures. The current distributions could be optimized by adjusting the radii of circular-shaped areas. Due to different arrangement of polarization conversion units and the different radius of circular-shaped area, the different current distributions are observed. However, same gain enhancement at 10.1 GHz is observed. It can be concluded that synthetical contribution of the induced current is made to enhance the radiation properties.

**Figure 8:**
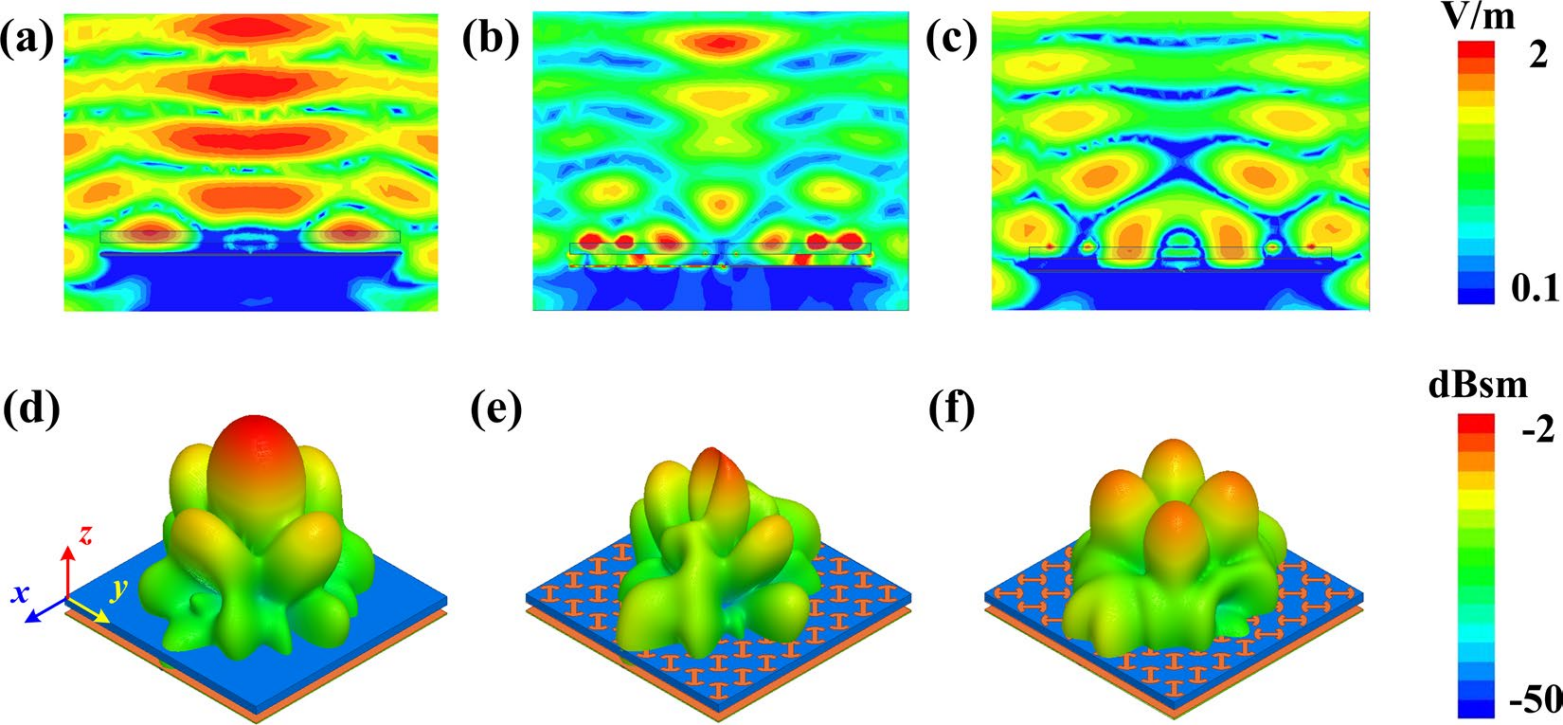
Electric fields and 3D scattering patterns of primary and proposed antennas. Electric fields of (**a**) primary antenna, (**b**) proposed PCS#1 antenna and (**c**) proposed PCS#2 antenna at 10.6 GHz. 3D scattering patterns of (**d**) primary antenna, (**e**) proposed PCS#1 antenna and (**f**) proposed PCS#2 antenna at 10.6 GHz.

To further verify scattering performance of proposed antennas, electric fields in *xoz* plane and 3D scattering patterns are presented in Fig. 8. As Fig. 8(a–c) shows, for normal incidence, the electric fields of proposed antennas at 10.6 GHz (one of the peak reduction frequency) in backward direction are weakened. These results indicate that incident waves are redirected to other angles. 3D scattering patterns of primary and proposed antennas are plotted in Fig. 8(d–f). For PCS#1 antenna, the strong back scattering is mainly redirected to *y*-axis and two dimples are observed in *x*-axis direction. However, for PCS#2 antenna, the main reflected lobes are dispersed to four quadrants, *φ* = 45°, 135°, 225° and 315°. The diverse electric fields and scattering patterns are attributed to the different scattering suppression mechanism.

### Fabrication and measurements

**Figure 9:**
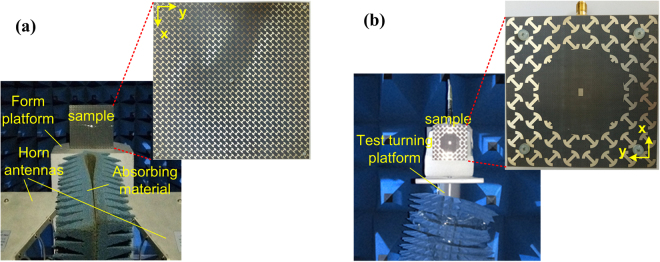
Fabricated samples and measurement circumstance and settings. (**a**) Polarization conversion MS. (**b**) Proposed PCS#2 antenna.

**Figure 10:**
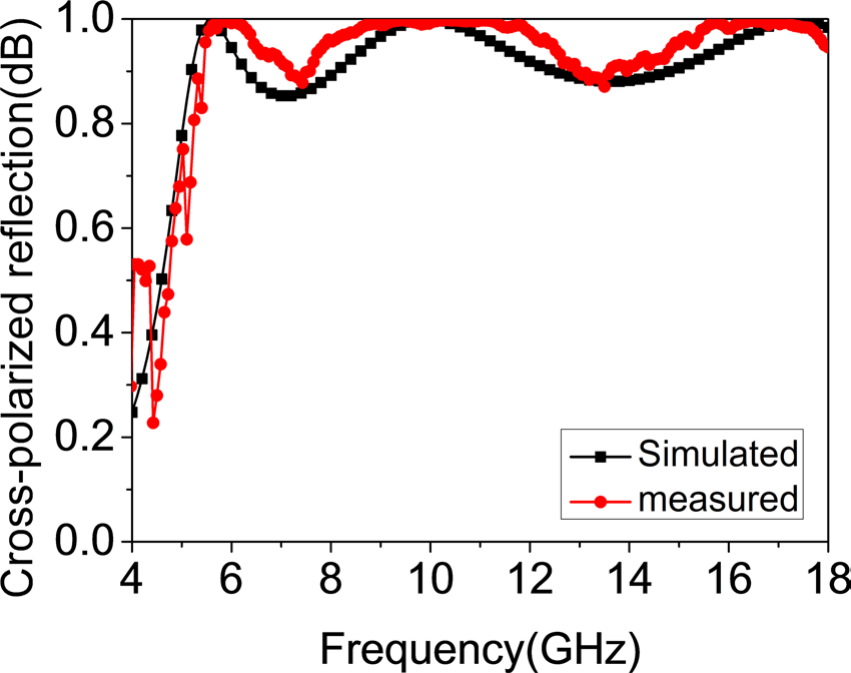
Measured cross-polarized reflection magnitudes.

To further validate the design method, the proposed polarization conversion MS and MS-based antennas were fabricated using the standard printed circuit board (PCB) technology and measured in the anechoic chamber, shown in Fig. 9(a,b). Nylon spacers are used to support the dielectric substrate above the ground plane, and then the air substrate is created. Because the two MS-based antennas possess radiation enhancement, we take the proposed PCS#2 antennas as an example for verification. For plane wave incidence cases, the polarization conversion MS is measured using free-space wave method^30^. As shown in Fig. 9(a), two broadband horn antennas connected to a vector network analyzer (VNA) Agilent N5230C are used to transmit the EM waves and receive the reflected waves. A piece of absorbing material is set between the antennas to reduce undesired coupling. In this way, the polarization conversion properties could be obtained from the measured S parameters. It is worthwhile to point out that the two horn antennas are set as *x*-polarization that is the same as the MS in simulations. The gate-reflect-line calibration in time-domain analysis kit of VNA is used to experimentally verify and improve the testing capability. Moreover, a metallic plate with the same aperture size of the MS is also measured for calibration. As Fig. 10 presents, the measured results of cross-polarized reflection coincide with the simulated predictions. The reflection magnitudes are more than 0.85 from 5.3 to 18.0 GHz. Due to the restrictions of test conditions, the reflectance of proposed polarization conversion MS is not measured in the frequency range higher than 18.0 GHz.

**Figure 11:**
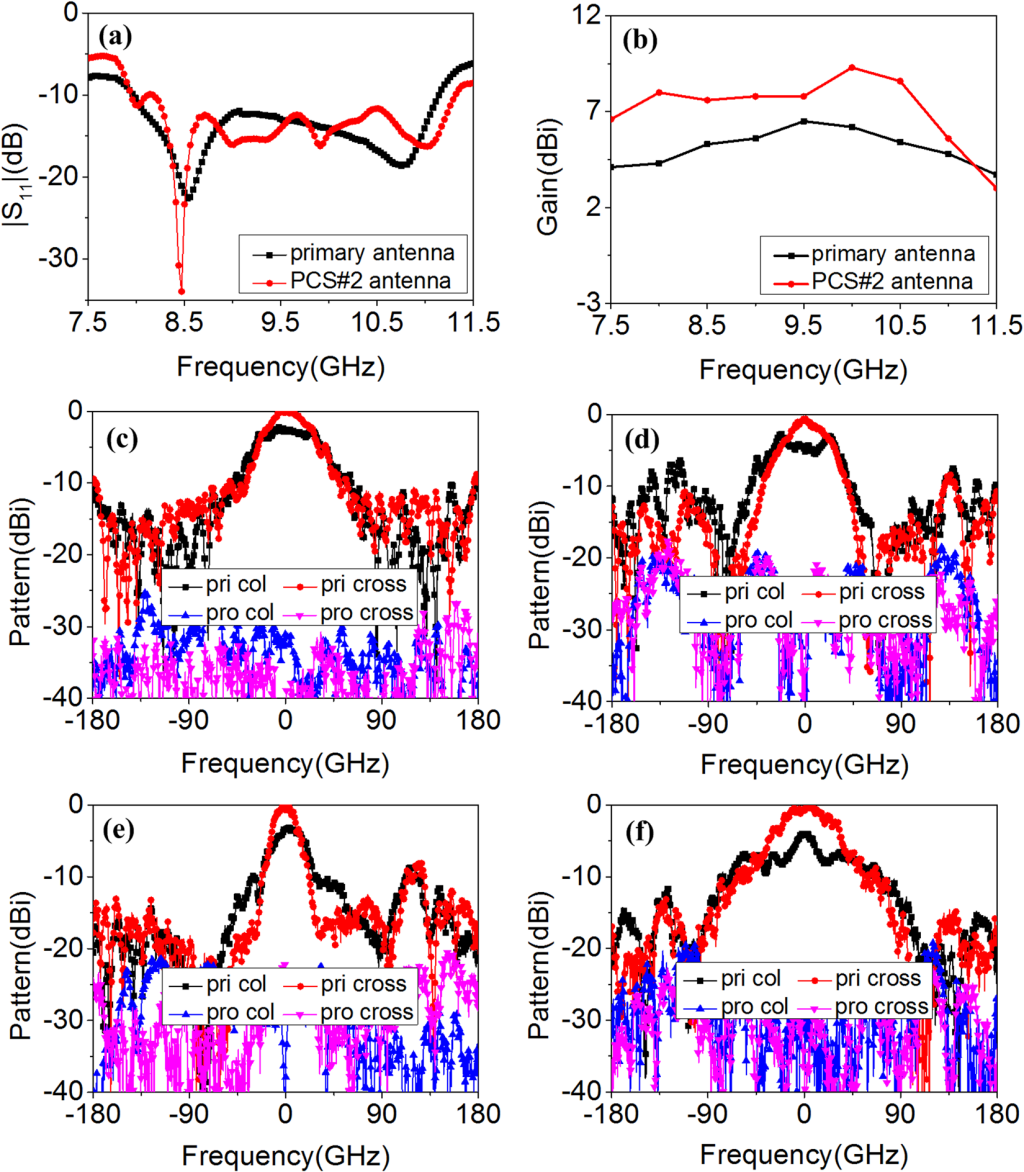
The measured radiation properties of proposed PCS#2 antenna. (**a**) S_11_, (**b**) Boresight gain, (**c**) *xoz* and (**d**) *yoz* plane radiation patterns at 8.5 GHz, (**e**) *xoz* and (**f**) *yoz* plane radiation patterns at 10.1 GHz.

**Figure 12:**
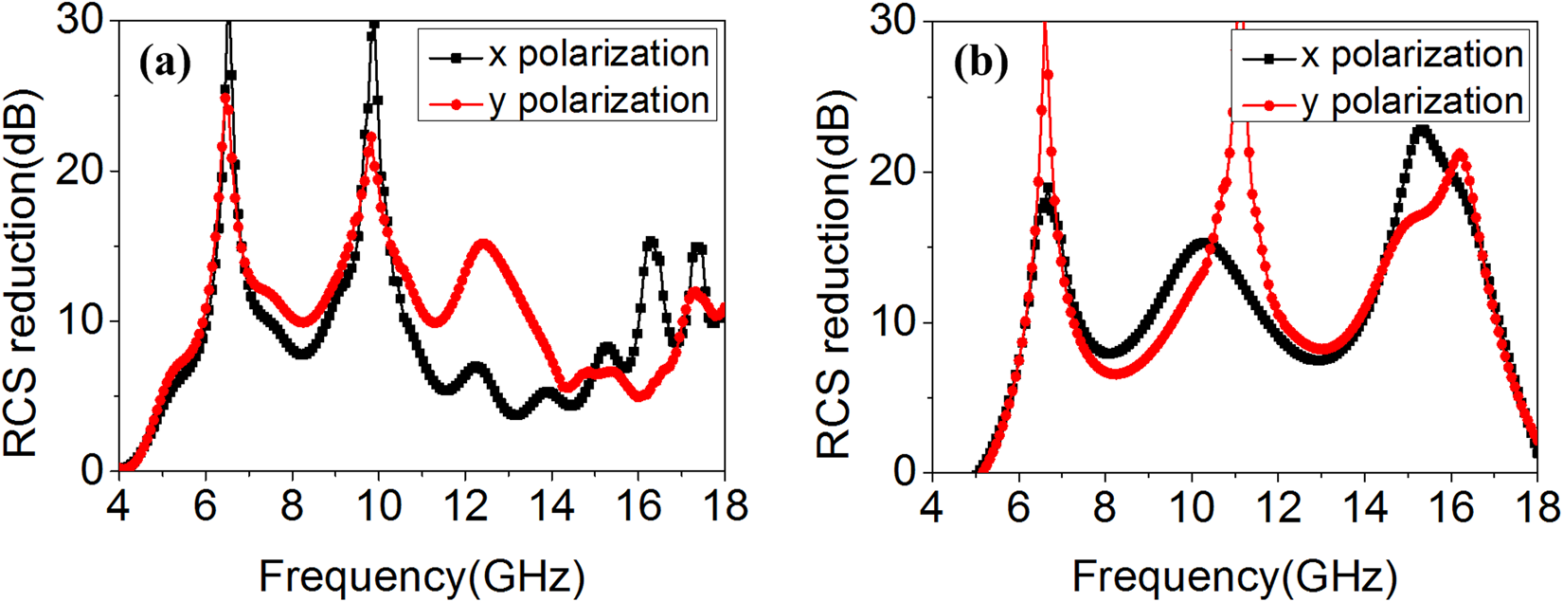
The measured RCS reduction of proposed PCS#2 antenna for vertical and horizontal polarizations. The incident angle (**a**) theta = 0°, (**b**) theta = 30°.

For the measurement of antenna radiation performance, the S_11_ is measured using the VNA. The gain and radiation patterns of the antennas are measured in the anechoic chamber to eliminate noise interference and the standard gain-comparison method is adopted^42^. Measurement circumstance and settings are also presented in Fig. 9(b). The reflection coefficient S_11_ below −10 dB is from 8.0 to 11.1 GHz for the primary antenna and from 8.0 to 11.3 GHz for the proposed one, shown in Fig. 11(a). The relative bandwidth of proposed antenna is 34.2%. The wide impedance bandwidth is preserved. The measured boresight gain is plotted in Fig. 11(b). The gain of proposed antenna is enhanced almost from 7.5 to 11.3 GHz, covering the operation band. The radiation patterns of primary and proposed antennas at 8.5 GHz and 10.1 GHz (the selection is random) are shown in Fig. 11(c–f). The main lobes of primary antenna are broad in *xoz* and *yoz* plane, whereas the proposed antenna produces highly directive beams. The peak radiation all happens in the broadside direction and obvious gain enhancement is observed at 8.5 GHz and 10.1 GHz. Moreover, the measured values of cross-polarization of proposed antenna are almost less than −20 dB. From the above results, it can be concluded that the proposed antenna achieve better radiation performance. The scattering measurement settings of the antennas are similar to these of polarization conversion MS. The primary and proposed antennas are terminated with matched load. The measured scattering performance is presented in Fig. 12(a,b). For normal incidence, the RCS is reduced from 4.0 to 18.0 GHz for vertical (*x* in simulation) and horizontal (*y* in simulation) polarization. The peak reduction reaches 30.0 dB. Meanwhile, for 30° oblique incidence, the specular RCS reduction is achieved from 5.0 to 18.0 GHz for arbitrary polarizations. From the above results, ultra-wideband low-scattering performance of the MS-based antenna is verified.

## Conclusions

An ultra-wideband polarization conversion MS and its two application cases are proposed and demonstrated. Considering the application on ACPA, air substrate and dielectric substrate are utilized to design this double-layer polarization conversion MS. And the polarization conversion bandwidth is broadened toward low-frequency range. Subsequently, the polarization conversion structures are used to construct uniform and orthogonal arrangement configurations based on different mechanism of scattering suppression. Two kinds of configurations are readily integrated into ACPA with coplanar loading method and MS-based antennas are formed. The wide operation band is preserved well and boresight gain is enhanced over the whole operation band. Meanwhile, the scattering of antennas is suppressed in an ultra-wide frequency range. The proposed MS-based ACPAs possess radiation enhancement and scattering suppression simultaneously. The contradiction between radiation and scattering is addressed well. The results demonstrate that we provide novel approach to design high-performance polarization conversion MS and MS-based devices.

## Methods

### Numerical simulations

The polarization conversion MS and the MS-based antennas are simulated in commercial software Ansoft HFSS. The unit cell with master-slave periodic boundaries and floquet port is used to mimic the periodically arranged polarization conversion MS. A plane wave under normal incidence is introduced to obtain scattering properties of MS-based antennas.

### Sample fabrications and measurements

The samples of the polarization conversion MS and the MS-based antennas are fabricated by using standard printed circuit board (PCB) technology. Nylon spacers are utilized to support dielectric substrate above ground plane. Thus, air substrate is created. The polarization conversion MS is measured using free-space wave method. Two broadband (1–18 GHz) horn antennas connected to a vector network analyzer (VNA) Agilent N5230C are used to transmit the EM waves and receive the reflected waves. The two antennas are set as *x*-polarization that is the same as the MS in simulations. A piece of absorbing material is set between the antennas to reduce undesired coupling. The gate-reflect-line calibration in time-domain analysis kit of VNA is used to experimentally verify and improve the testing capability. Moreover, a metallic plate with the same aperture size of the MS is also measured for calibration. In this way, the polarization conversion properties could be obtained from the measured S parameters. For the measurements of radiation performance of antennas, S_11_ is measured by using the VNA. The gain and radiation patterns of antennas are measured in the anechoic chamber and the standard gain-comparison method is adopted. The scattering measurements of antennas are similar to these of polarization conversion MS. The antennas are terminated with matched load. As for the oblique incidence case, the two horn antennas are set as x-polarization and deviated from the normal direction with an opposite specular angle.
